# Obese dogs exhibit different fecal microbiome and specific microbial networks compared with normal weight dogs

**DOI:** 10.1038/s41598-023-27846-3

**Published:** 2023-01-13

**Authors:** Hanbeen Kim, Jakyeom Seo, Tansol Park, Kangmin Seo, Hyun-Woo Cho, Ju Lan Chun, Ki Hyun Kim

**Affiliations:** 1 Department of Animal Science, Life and Industry Convergence Research Institute, Pusan National University, Miryang, 50463 Republic of Korea; 2 Department of Animal Science and Technology, Chung-Ang University, Anseong-si, Gyeonggi-Do 17546 Republic of Korea; 3 Animal Welfare Research Team, National Institute of Animal Science, Wanju-gun, 55365 Republic of Korea

**Keywords:** Microbiology, Physiology

## Abstract

Canine obesity is a major health concern that predisposes dogs to various disorders. The fecal microbiome has been attracting attention because of their impact on energy efficiency and metabolic disorders of host. However, little is known about specific microbial interactions, and how these may be affected by obesity in dogs. The objective of this study was to investigate the differences in fecal microbiome and specific microbial networks between obese and normal dogs. A total of 20 beagle dogs (males = 12, body weight [BW]: 10.5 ± 1.08 kg; females = 8, BW: 11.3 ± 1.71 kg; all 2-year-old) were fed to meet the maintenance energy requirements for 18 weeks. Then, 12 beagle dogs were selected based on body condition score (BCS) and divided into two groups: high BCS group (HBCS; BCS range: 7–9, males = 4, females = 2) and normal BCS group (NBCS; BCS range: 4–6, males = 4, females = 2). In the final week of the experiment, fecal samples were collected directly from the rectum, before breakfast, for analyzing the fecal microbiome using 16S rRNA gene amplicon sequencing. The HBCS group had a significantly higher final BW than the NBCS group (*P* < 0.01). The relative abundances of *Faecalibacterium*, *Phascolarctobacterium*, *Megamonas*, *Bacteroides*, *Mucispirillum*, and an unclassified genus within *Ruminococcaceae* were significantly higher in the HBCS group than those in the NBCS group (*P* < 0.05). Furthermore, some Kyoto Encyclopedia of Genes and Genomes (KEGG) modules related to amino acid biosynthesis and B vitamins biosynthesis were enriched in the HBCS group (*P* < 0.10), whereas those related to carbohydrate metabolism were enriched in the NBCS group (*P* < 0.10). Microbial network analysis revealed distinct co-occurrence and mutually exclusive interactions between the HBCS and NBCS groups. In conclusion, several genera related to short-chain fatty acid production were enriched in the HBCS group. The enriched KEGG modules in the HBCS group enhanced energy efficiency through cross-feeding between auxotrophs and prototrophs. However, further studies are needed to investigate how specific networks can be interpreted in the context of fermentation characteristics in the lower gut and obesity in dogs.

## Introduction

Owing to the growing interest in dog welfare, more people have started to take an interest in the health problems of dogs^1^. Among them, canine obesity is considered a major metabolic disease because it can predispose dogs to a variety of disorders, such as cardiovascular disease^2^, expiratory airway dysfunction^3^, and diabetes mellitus^4^, and has detrimental effects on longevity^5^. The main cause of obesity in dogs is an imbalance between energy intake and expenditure^6^. Furthermore, some studies suggest that breed, feed quality, and the owner’s interest in maintaining pet’s health are also possible causes^7–9^. Therefore, the veterinary community is paying increasing attention to the prevention of obesity in dogs.

Previously, companion dogs have been evaluated for being overweight and obese based on their body weight (BW), body condition score (BCS), and body composition (fat mass and lean body mass)^10–12^. These methods have been recognized to be efficient in terms of cost and convenience. However, given the limited data on the ideal weight for each breed and the subjective opinions of various veterinarians, these methods need to be improved for better reliability^6^. Accordingly, recent advances in omics technology are expected to replace traditional methods and provide new methods for investigating obesity indicators.

The gastrointestinal tract of dogs, especially the colon, is habituated by a complex microbiota that contributes to energy source absorption, metabolic functions, and immunogenic responses^13^. Several studies have investigated changes in the fecal microbiota in response to the modulation of dietary components, including carbohydrates^14^, proteins^15^, and fats^16^. In addition, some studies have reported differences in the fecal microbiota of obese dogs after a weight-loss program^17–20^. Taken together, these studies highlight the importance of the fecal microbiota in sustaining canine health and controlling obesity.

Previous studies have also reported differences in the gut microbiota among obese and lean individuals in humans and mice^21–25^. These changes are characterized by a reduced diversity of gut microbiota^21,22^, increased *Firmicutes*/*Bacteroidota* ratio^23,24^, and better energy harvesting^25^ in the obese group.

However, only a few studies have investigated the differences in the fecal microbiota between obese and lean dogs^17,20,26^. The composition of the gut microbiota in dogs can differ based on several factors, including diet, age, breed, and disorders^27–29^. Previous studies had several limitations in interpreting the relationship between obesity and gut microbiota, including the lack of unification in terms of breeds or ages^17,20,26^. Therefore, the present study investigated the effects of obesity on the gut microbiota and microbial functional features of dogs of a similar age and breed. We hypothesized that fecal microbiota, microbial functional profiles, and networks among microbiota would differ between obese and normal dogs.

Therefore, this study aimed to investigate differences in fecal microbiota composition, microbial functional profiles, and specific networks between obese and normal BCS groups after maintenance energy requirements were met.

## Methods

### Animals and experimental design

This study was conducted using neutered beagles owned by the National Institute of Animal Science in Korea. This experiment was conducted in accordance with the methods approved by the Animal Care and Use Committee National Institute of Animal (NIAS-2019-370). All methods for animal care are performed in accordance with the ARRIVE guidelines. Twenty beagle dogs (males = 12, initial body weight [BW]: 10.5 ± 1.08 kg; females = 8, initial BW: 11.3 ± 1.71 kg; all 2-year-old) were enrolled in this study. The beagles lived individually in an indoor space (1.8 m × 2.6 m) maintained at a temperature of 22–24 °C and relative humidity from 60 to 80% using automatic temperature control and continuous ventilation. All dogs were fed twice a day (at 10:00 and 16:00), had access to drinking water ad libitum, and engaged in approximately 3 h of outdoor activity every day. During the outdoor activity, all dogs were in the outdoor playground (1.8 m × 2.6 m), individually. In the outdoor playground, the floor was tiled and the side walls were bricked. One side was made of a stainless fence that allows the dog to see outside. All dogs did not have access to any grass, soil, or plant, and freely come and go indoor and outdoor environments during the outdoor activity. The dogs were fed an extruded pellet-type commercial dry food (Iskhan All-life33®; Wooriwa Ltd., Korea) that met the maintenance energy requirements estimated based on the formula suggested by the Association of American Feed Control Officials (AAFCO; metabolizable energy requirement = 132 kcal × kg BW^0.75^/day)^30^ (Table 1). The experiment was conducted for 18 weeks. In the first and final day of the experiment, the BCS was measured on a nine-point scale according to the criteria of Laflamme D^12^, and 12 neutered beagles (eight males and four females, all 2-years-old) were selected based on the final BCS 24 h before collecting feces and divided into two groups, each consisting of four males and two females. The normal BCS group (NBCS) was composed of six healthy dogs with a normal BCS (4–6), and the high BCS group (HBCS) was composed of six dogs with a high BCS (7–9) (Table 2).

**Table 1:** Analyzed chemical composition of the experimental diet.

Chemical composition (%DM or as stated)	
DM, %	88.0
CP	37.5
EE	22.7
CF	3.41
NFE^1^	21.6
Ash	14.8
Ca	1.00
P	0.80
ME^2^, Mcal/kg	3.68

DM, dry matter; CP, crude protein; EE, ether extract; CF, crude fiber; NFE, nitrogen-free extract; Ca, calcium; P, phosphorus; ME, metabolizable energy.

^1^NFE% = 100 − (CP + EE + CF + Ash + moisture).

^2^Estimated based on National Research Council^87^.

**Table 2:** Differences in body weight and body condition score between obese and normal dogs.

Parameters	Treatment^1^			
	HBCS	NBCS	SEM	*P-*value
Initial BW, kg	11.88	10.05	0.590	0.0261
Final BW, kg	13.10	10.07	0.772	0.0045
BW gain, kg	1.22	0.02	0.375	0.0287
Initial BCS	7.0	5.2	0.601	0.0262
Final BCS	8.0	5.0	0.365	< 0.0001

SEM, standard error of the mean.

^1^HBCS, high body condition score (BCS) group (BCS range: 7–9, 4 males and 2 females, all 2-year-old); NBCS, normal BCS group (BCS range: 4–6, 4 males and 2 females, all 2-year-old).

### Fecal sample collection and DNA extraction

Twenty-four hours before collecting feces, feeding was restricted. To avoid possible contamination and minimize external exposure, fecal samples were collected directly from the rectum using cotton swabs before breakfast (9:00 to 10:00), immersed in liquid nitrogen, and stored in a deep freezer at − 80 °C until analysis. DNA was extracted from the fecal samples using the NucleoSpin DNA Stool kit (Macherey–Nagel, Germany) according to the manufacturer’s instructions. Briefly, fecal samples (180–220 mg) were lysed using the MN Bead Tube Holder, and the genomic DNA was harvested through NucleoSpin DNA stool column centrifugation at 13,000 × *g* for 1 min. In the final step, genomic DNA was eluted into the elution buffer after centrifugation at 13,000 × *g* for 1 min and stored at − 20 °C.

### 16S rRNA gene amplicon sequencing and data processing

For PCR amplification targeting the V3 and V4 variable regions of 16S rRNA, the following primer sets were used^31^: forward primer 5′-TCG TCG GCA GCG TCA GAT GTG TAT AAG AGA CAG CCT ACG GGN GGC WGC AG-3′ and reverse primer 5′-GTC TCG TGG GCT CGG AGA TGT GTA TAA GAG ACA GGA CTA CHV GGG TAT CTA ATC C-3′. Illumina adapter overhang nucleotide sequences were added to the gene-specific sequences. The locus-specific sequences were as follows: forward overhang 5′-TCG TCG GCA GCG TCA GAT GTG TAT AAG AGA CAG-3′ and reverse overhang 5′-GTC TCG TGG GCT CGG AGA TGT GTA TAA GAG ACA G-3′. To purify the PCR products, KAPA HiFi HotStart ReadyMix (KAPA Biosystems, USA) and Agencourt AMPure XP system (Beckman Coulter Genomics, USA) were used. The libraries were sequenced using the Illumina MiSeq instrument (2 × 300 paired-end sequencing; Illumina, CA, USA).

The barcode sequences were trimmed using Cutadapt (version 1.11)^32^. Then, the raw amplicon sequences were analyzed using built-in plugins within Quantitative Insights into Microbial Ecology 2 (QIIME2) (version 2021.02)^33^. First, the DADA2 plugin was used to remove primer sequences, denoise low-quality reads (Q < 25), merge forward and reverse reads, and remove chimeric sequences. The raw 16S rRNA gene sequences are available in the National Center for Biotechnology Information sequence read archive (BioProject accession: PRJNA818097). The amplicon sequence variants (ASVs) were taxonomically classified using the Silva 16S rRNA gene database (version SSU138)^34^. Taxonomically unassigned ASVs were excluded. The alpha diversity of each sample was examined with respect to ACE (abundance-based coverage estimator) and Chao1 estimates, observed ASVs, Pielou's evenness index, Shannon diversity index, and Simpson diversity index based on rarefied ASV tables using randomly selected 67,151 ASVs per sample. Principal coordinate analysis (PCoA) was conducted based on unweighted and weighted UniFrac distance matrices to compare the overall dissimilarity of microbiota between the HBCS and NBCS groups and visualized using Emperor implemented in QIIME2. The number of common and exclusively identified bacterial taxa between the HBCS and NBCS groups at the phylum, family, and genus levels were visualized using Venn diagrams. Functional metagenomic features from the 16S rRNA gene sequences were predicted based on ASVs and the corresponding biological observation matrix (BIOM) table using the Phylogenetic Investigation of Communities by Reconstruction of Unobserved State (PICRUSt2) (version 2.3.0-b) with default options^35^. Principal component analysis (PCA) was performed to compare the overall differences in predicted functional profiles based on the Kyoto Encyclopedia of Genes and Genomes (KEGG) orthologs between the HBCS and NBCS groups. The PCA plot was visualized using the ggfortify R package^36^. FastSpar was used to examine the correlations among the major bacterial genera in the HBCS and NBCS groups^37^, and the correlations were computed based on the sparse correlations for compositional data^38^. Only significant microbial interactions (*P* < 0.05) were further analyzed using co-expression differential network analysis (CoDiNA)^39^ to identify specific microbial networks in the HBCS and NBCS groups.

### Statistical analysis

Before statistical analysis, all data were tested for normal distribution using the Shapiro–Wilk test in SAS 9.4 (SAS Institute Inc., NC, USA). Then, normally distributed data, including those on animal performance (BW, BCS, and BW gain) and alpha diversity indices (ACE, Chao1, observed ASVs, Pielou's evenness index, Shannon index, and Simpson diversity index) were analyzed using a *t*-test in SAS 9.4. Data that were not normally distributed, including major microbial taxa and predicted microbial functional features from KEGG categories and modules, were analyzed using a non-parametric Wilcoxon rank-sum test. Permutational multivariate analysis of variance (PERMANOVA) was used to further analyze the PCoA and PCA results to assess whether the overall microbial community and predicted functional profiles (KEGG orthologs) differed significantly between the HBCS and NBCS groups. Statistical significance was declared at *P* < 0.05, and a statistical trend was speculated at 0.05 ≤ *P* ≤ 0.10.

### Ethical approval and consent to participate

The animal study was reviewed and approved by the National Institute of Animal Science in Korea**.** This experiment was conducted in accordance with the methods approved by the Animal Care and Use Committee National Institute of Animal (NIAS-2019-370).

## Results

### Relation between BCS and animal performance

Both the initial BW and BCS were higher in the HBCS group than those in the NBCS group (Table 2). Even both HBCS and NBCS groups fed maintenance energy requirements to maintain stable weight, the HBCS group had a significantly higher final BW and BCS than the NBCS group (*P* < 0.05), resulting in a higher BW gain in the HBCS group (Table 2).

### Quality assessment and sample statistics

A total of 1,826,502 sequences were obtained from 16S rRNA amplicon sequencing analysis, with an average of 152,209 ± 30,573 sequences per fecal sample (Table S1). After quality filtering using QIIME 2 (Q score > 25), 1,193,079 sequences (65% of the raw reads) were generated, with an average of 99,423 ± 19,814 sequences per sample. Good’s coverage was greater than 99% for all fecal samples.

### Relation between BCS and the alpha and beta diversity of fecal microbiota

**Figure 1:**
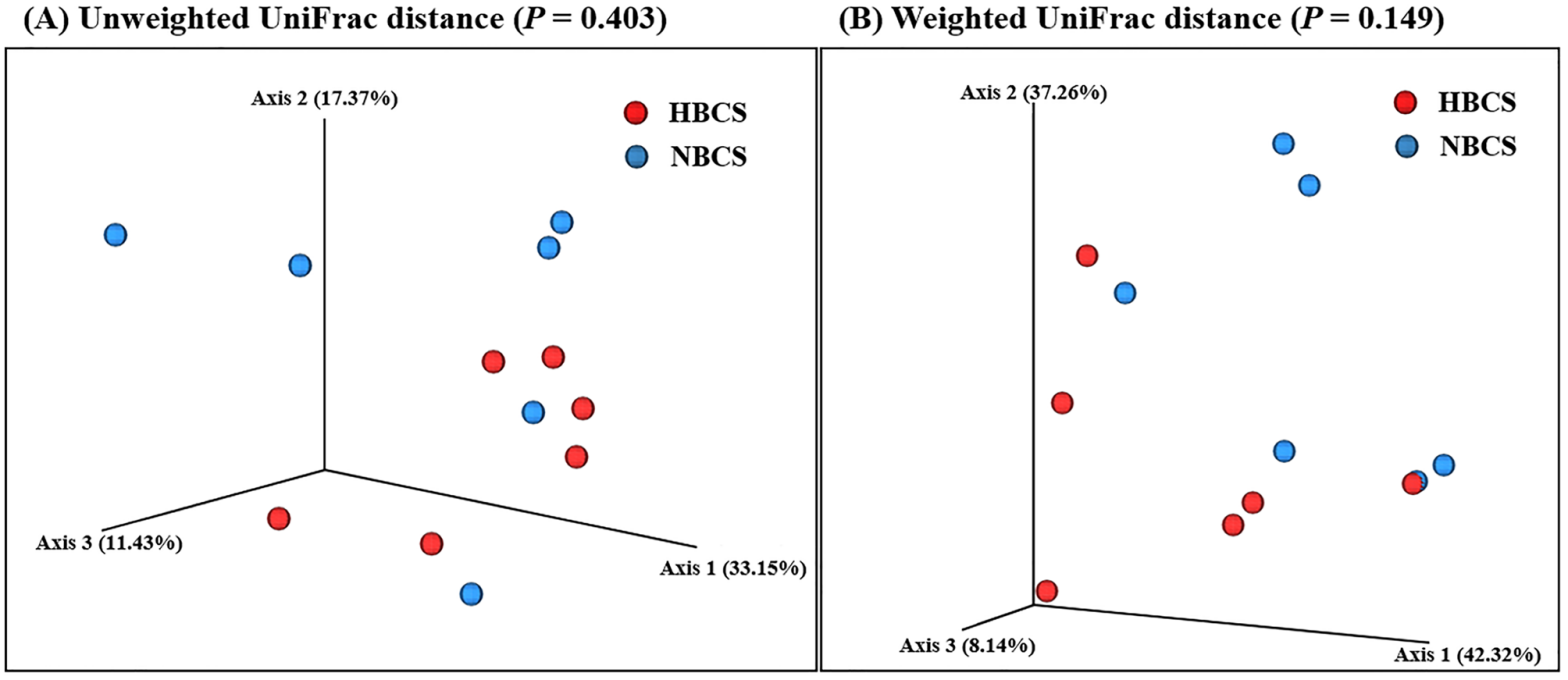
Principal coordinates analysis based on (**A**) unweighted UniFrac and (**B**) weighted UniFrac distance matrices of the fecal microbiota. Permutational multivariate analysis of variance was used to compare differences in fecal microbiota composition between high and normal body condition score (BCS) groups. HBCS, high BCS group (BCS range: 7–9, 4 males and 2 females, all 2-year-old); NBCS, normal BCS group (BCS range: 4–6, 4 males and 2 females, all 2-year-old).

In fecal microbiota, the difference in BCS did not result in significant changes in any of the alpha diversity measurements (Table 3). The PCoA results based on both unweighted and weighted UniFrac distances also did not present any significant difference in the fecal microbiota of the HBCS and NBCS groups (Fig. 1).

**Table 3:** Differences in alpha diversity measurements of the fecal microbiota by obesity.

Parameters	Treatments^1^			
	HBCS	NBCS	SEM^2^	*P-*value
Observed ASVs	180	174	19.1	0.8220
Chao1 estimates	181	175	19.5	0.8268
ACE	180	175	19.4	0.8344
Pielou's evenness index	0.698	0.636	0.0261	0.1044
Shannon’s index	5.22	4.72	0.251	0.1830
Simpson’s index	0.934	0.897	0.0181	0.1361

ASV, amplicon sequencing variant; ACE, abundance-based coverage estimator.

^1^HBCS, high body condition score (BCS) group (BCS range: 7–9, 4 males and 2 females, all 2-year-old); NBCS, normal BCS group (BCS range: 4–6, 4 males and 2 females, all 2-year-old).

^2^SEM, standard error of the mean.

### Relation between BCS and the fecal microbiota

In this study, only bacterial taxa have been presented because archaeal reads could not be detected. The bacterial taxa that were detected in over 50% of the samples and had a relative abundance of over 0.05% in at least one of the BCS groups were defined as major microbial taxa.

**Figure 2:**
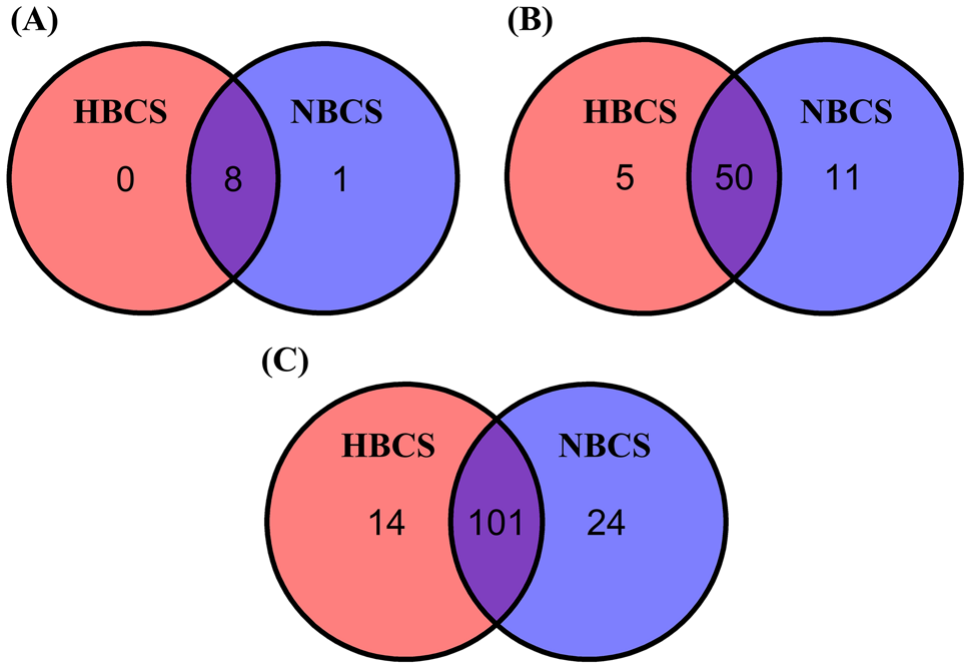
Venn diagram of common and exclusively identified bacterial taxa in collapsed (**A**) phyla, (**B**) families, and (**C**) genera based on biological observation matrix (BIOM) tables between high and normal body condition score (BCS) groups. HBCS, high BCS group (BCS range: 7–9, 4 males and 2 females, all 2-year-old); NBCS, normal BCS group (BCS range: 4–6, 4 males and 2 females, all 2-year-old).

The Venn diagram shows common and exclusively identified prokaryotic taxa in the HBCS and NBCS groups (Fig. 2). Eight of the nine phyla were shared between the HBCS and NBCS groups, and only *Desulfobacterota* was exclusively detected in the NBCS group (Fig. 2A); moreover, *Desulfobacterota* was detected in only one of the six samples. At the family level, 50 families (approximately 75.8%) were shared between the HBCS and NBCS groups and no major families were found exclusively in the BCS group (Fig. 2B). At the genus level, more than 100 genera (approximately 72.7%) were shared between the HBCS and NBCS groups, and only 14 and 24 genera were exclusively detected in the HBCS and NBCS groups, respectively (Fig. 2C). *Succinivibrio* and an unclassified genus within *Atopobiaceae* were the only major genera (present in at least 50% of the samples in each group) found exclusively in the HBCS and NBCS groups, respectively; however, they only accounted for a minor proportion (relative abundance < 0.01%).

**Figure 3:**
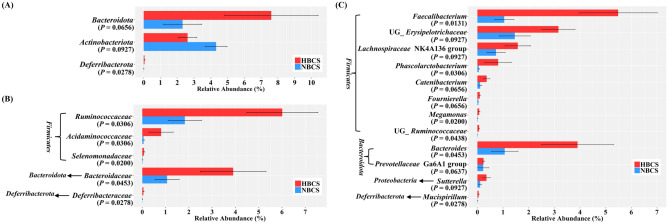
Differences in fecal microbiota at (**A**) phylum, (**B**) family, and (**C**) genus levels (each representing ≥ 0.05% in at least one of the treatments). Taxa representing at least statistical tendency (*P* < 0.10) are presented. HBCS, high BCS group (BCS range: 7–9, 4 males and 2 females, all 2-year-old); NBCS, normal BCS group (BCS range: 4–6, 4 males and 2 females, all 2-year-old).

The major phyla, families, and genera with relative abundances of over 0.5% in at least one group are shown in Figure S1. Among them, the major taxa that showed statistical tendency (*P* < 0.10) are shown in Fig. 3 (relative abundance ≥ 0.05% in at least one group). *Firmicutes* was the most predominant phylum (accounting for at least 77% of the total microbiota), followed by *Fusobacteriota* (Figure S1A). Of the five predominant phyla, the relative abundance of *Bacteroidota* tended to be higher in the HBCS group than that in the NBCS group (HBCS, 7.61% vs. NBCS, 2.32%; *P* = 0.0656), whereas *Actinobacteria* showed an opposite trend (HBCS, 2.62% vs. NBCS, 4.32%; *P* = 0.0927) (Fig. 3A). The relative abundance of *Deferribacterota*, accounting for a minor proportion, was significantly higher in the HBCS group than that in the NBCS group (HBCS, 0.051% vs. NBCS, 0.008%; *P* = 0.0278) (Fig. 3A). At the family level, *Lachnospiraceae*, *Peptostreptococcaceae*, and *Lactobacillaceae* were the three most abundant families in the HBCS group, whereas *Peptostreptococcaceae*, *Erysipelotrichaceae*, and *Lactobacillaceae* were the most abundant in the NBCS group (Figure S1B). Among the major classified families, the relative abundances of *Ruminococcaceae*, *Bacteroidaceae*, *Acidaminococcaceae*, *Selenomonadaceae*, and *Deferribacteraceae* were higher in the HBCS group than those in the NBCS group (Fig. 3B). At the genus level, *Peptoclostridium* was the most predominant genus in both BCS groups (HBCS, 14.90% vs. NBCS, 23.79%), and more than 84% of the entire fecal microbiota was assigned to 54 major classified genera (representing > 0.05% of relative abundance in at least one BCS group). At the genus level, the relative abundances of *Bacteroides, Mucispirillum,* and the four genera within *Firmicutes* (*Faecalibacterium*, *Phascolarctobacterium*, *Megamonas,* and UG_*Ruminococcaceae*) were significantly higher in the HBCS group than those in the NBCS group (Fig. 3C, *P* < 0.05). Meanwhile, the relative abundances of *Sutterella*, *Prevotellaceae* Ga6A1 group, and four genera within *Firmicutes* (UG_*Erysipelotrichaceae*, *Lachnospiraceae* NK4A136 group, *Catenibacterium,* and *Fournierella*) tended to be higher in the HBCS group than those in the NBCS group (Fig. 3C, *P* < 0.10).

### Relation between BCS and the predicted functional profiles from fecal microbiota

**Figure 4:**
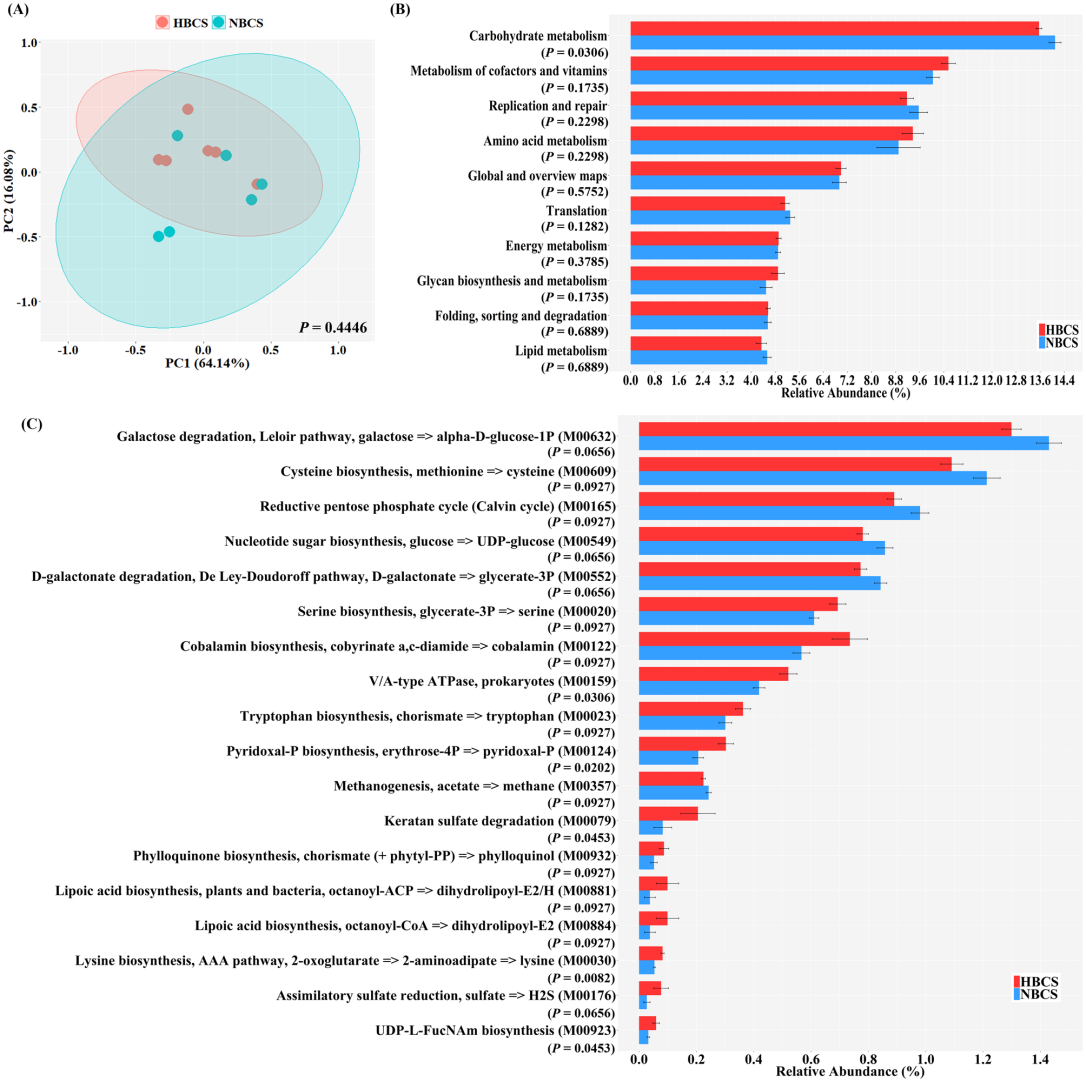
Differences in predicted functional profiles of fecal microbiota. (**A**) Principal component analysis of Kyoto Encyclopedia of Genes and Genomes (KEGG) orthologs^89^. (**B**) Distribution of top 10 KEGG categories. (**C**) Distribution of major KEGG modules (of which relative abundances were over 0.05% in at least one group) showing at least statistical tendency (*P* < 0.10). All microbial functional profiles were predicted using Phylogenetic Investigation of Communities by Reconstruction of Unobserved States version 2.0. HBCS, high BCS group (BCS range: 7–9, 4 males and 2 females, all 2-year-old); NBCS, normal BCS group (BCS range: 4–6, 4 males and 2 females, all 2-year-old).

The overall distribution of the microbial functional profiles predicted based on KEGG orthologs was visualized using PCA plots (Fig. 4A). There was no significant difference in the overall distribution of KEGG orthologs between the HBCS and NBCS groups based on the PERMANOVA analysis. Among the top 10 KEGG categories (Level 2), only carbohydrate metabolism was significantly higher in the NBCS group than that in the HBCS group (Fig. 4B, *P* < 0.05). Among the major KEGG modules, three modules related to amino acid metabolism (lysine biosynthesis [M00030], serine biosynthesis [M00020], and tryptophan biosynthesis [M00023]) and five modules related to the metabolism of cofactors and vitamins (cobalamin biosynthesis [M00122], pyridoxal-P biosynthesis [M00124], phylloquinone biosynthesis [M00932], and lipoic acid biosynthesis [M00881, M00884]) were markedly enriched in the HBCS group (Fig. 4B, *P* < 0.10). In contrast, three modules related to carbohydrate metabolism (galactose degradation [M00632], nucleotide sugar biosynthesis [M00549], and D-galactonate degradation [M00552]), two modules related to energy metabolism (reductive pentose phosphate cycle [M00165] and methanogenesis [M00357]), and one related to cysteine biosynthesis [M00609] tended to be higher in the NBCS group (Fig. 4C, *P* < 0.10).

### Differential network analysis based on BCS

**Figure 5:**
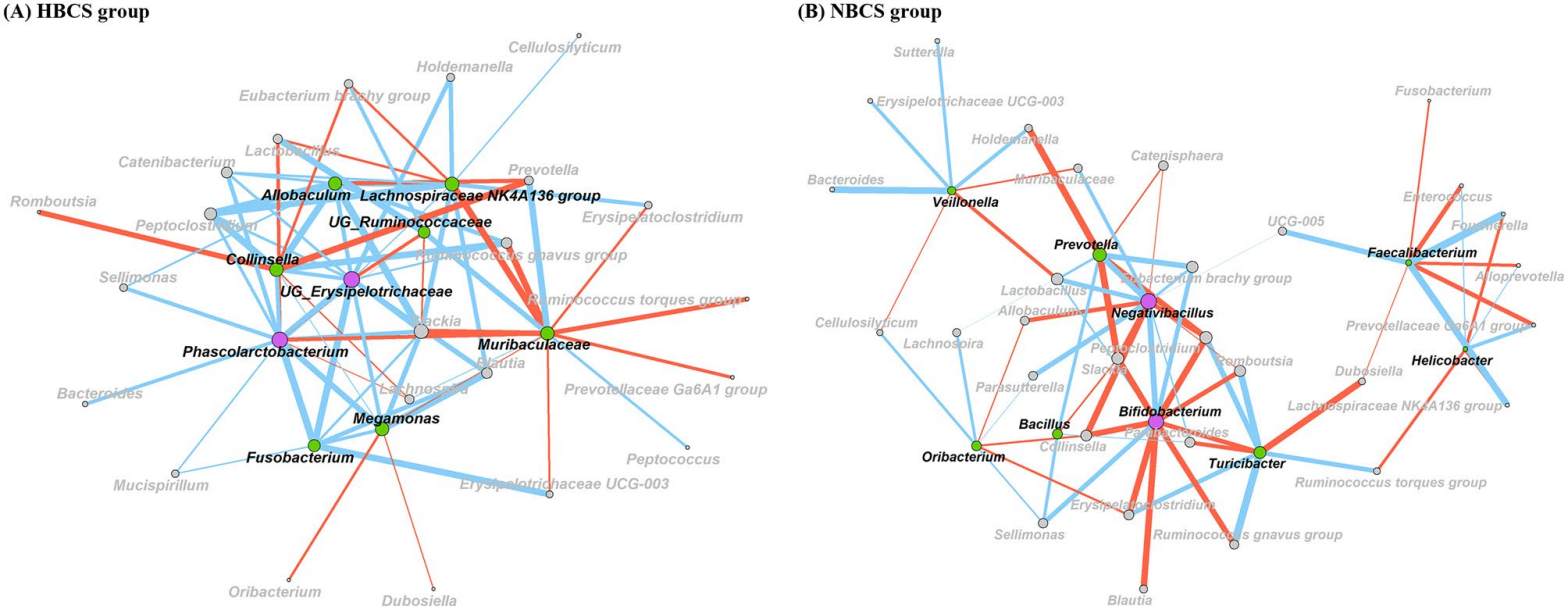
Exclusive co-occurrence and mutual exclusion networks in (**A**) high body condition score group (HBCS; BCS range: 7–9, 4 males and 2 females, all 2-year-old) and (**B**) normal BCS group (NBCS; BCS range: 4–6, 4 males and 2 females, all 2-year-old). Only genera accounting for ≥ 0.05% abundance in at least one of the treatments were used. Node color represents exclusive microbial nodes (green) and keystone genera (magenta). Keystone genera were detected based on the hub score, degree centrality, and eigenvector centrality measurements. Edge color represents co-occurrence (blue) or mutually exclusive (red) interactions. Edge thickness was adjusted based on the absolute value of the correlation coefficients. UG, unclassified genus.

The statistics for the differential networks of the major genera in the HBCS and NBCS groups are listed in Table 4. The total number of nodes for the HBCS and NBCS groups was 31 and 39, respectively, and the total number of edges was 74 and 71, respectively. In the differential network of the HBCS group, positive edges were detected more frequently than negative edges (Table 4; 51 positive edges vs. 23 negative edges), whereas the number of positive edges was similar to that of negative edges in the differential network of the NBCS group (Table 4; 36 positive edges vs. 35 negative edges). In the differential networks of each treatment, the γ link, which was specific to one of the networks, was assigned to 29% of the HBCS group and 23.1% of the NBCS group (Table 4). The specific genera (γ link) in the differential network of the HBCS group were *Allobaculum*, *Lachnospiraceae* NK4A136 group, UG_*Ruminococcaceae*, *Collinsella*, UG_*Erysipelotrichaceae*, *Phascolarctobacterium*, *Muribaculaceae*, *Megamonas,* and *Fusobacterium* (Fig. 5A), and those of NBCS group were *Veillonella*, *Prevotella*, *Negativibacillus*, *Bifidobacterium*, *Bacillus*, *Oribacterium*, *Turicibacter*, *Faecalibacterium*, and *Helicobacter* (Fig. 5B). Based on the two centrality measurements (degree and eigenvector centrality) and hub score within each differential network, UG_*Erysipelotrichaceae* and *Phascolarctobacterium* were the keystone genera in HBCS, while *Negativibacillus* and *Bifidobacterium* were those in the NBCS group (Fig. 5 and Figure S2).

**Table 4:** Exclusive network statistics of fecal microbiota between obese and normal dogs.

Parameters	Treatments^1^	
	HBCS	NBCS
Total nodes	31	39
Total edges	74	71
Positive edges	51	36
Negative edges	23	35
Exclusive nodes (%)	29.0	23.1
Network diameter	4	6
Network density	0.159	0.096
Modularity^2^	0.258	0.444
Cluster	5	5
Clustering coefficient	0.289	0.132

^1^HBCS, high body condition score (BCS) group (BCS range: 7–9, 4 males and 2 females, all 2-year-old); NBCS, normal BCS group (BCS range: 4–6, 4 males and 2 females, all 2-year-old).

^2^Modularity calculated based on the Louvain method^88^.

## Discussion

Several human obesity studies have reported that obesity is generally linked to altered gut microbiota, presenting lower diversity in obese humans than that in lean humans^21,22^. In addition, a recent metagenomic study revealed that the dog microbiota was similar to human microbiota in comparison with pig or mouse microbiota, and the alteration of microbiota in response to dietary changes was also in agreement with that observed in human studies^40^. Therefore, we expected the HBCS group to have a lower microbiota diversity than the NBCS group. However, the present study revealed no significant differences in alpha diversity indices explaining richness (observed ASVs, Chao1, ACE), evenness, and overall diversity represented by Shannon's index and Simpson’s index between the HBCS and NBCS groups. In addition, there was no significant difference in beta diversity measurements between the HBCS and NBCS groups. Similar to the results of microbial diversity in this study, Handl et al.^17^ reported no significant differences in alpha diversity (Shannon’s index, Pielou's evenness index, and the number of operational taxonomic units, i.e. OTUs) and beta diversity between lean and obese companion dogs. In addition, a recent study that evaluated differences in the fecal microbiota among obese, lean, and weight loss dogs showed that there were no significant differences in alpha diversity (Faith’s phylogenetic diversity, Shannon’s index, and the number of OTUs) and beta diversity, whereas the Pielou's evenness index was significantly higher in the obese group than that in the lean and weight loss groups^20^.

Previous studies have reported that the five predominant phyla in the fecal microbiota of dogs are *Firmicutes*, *Bacteroidota*, *Proteobacteria*, *Actinobacteriota*, and *Fusobacterium*^27,40^ as shown in the present study. The *Firmicutes*/*Bacteroidota* ratio is generally considered an indicator of obesity because several studies in humans and mice have shown that the obese group has a higher *Firmicutes*/*Bacteroidota* ratio than the lean group^23,24^. In addition, a previous study proposed that *Firmicutes* was more effective at harvesting energy from a diet than *Bacteroidetes*; therefore, a higher *Firmicutes*/*Bacteroidota* ratio may promote weight gain^41^. However, in the present study, the relative abundance of *Bacteroidota* tended to be higher in the HBCS group, whereas there was no significant difference in the relative abundance of Firmicutes, thereby reducing the *Firmicutes*/*Bacteroidota* ratio in the HBCS group (data not shown). Meanwhile, some studies on gut microbiota composition in obese humans or mice have also reported conflicting results, such as a lower*Firmicutes*/*Bacteroidota* ratio^42^ or no difference in the *Firmicutes*/*Bacteroidota* ratio^43^. In addition, several studies have observed no significant differences in both *Firmicutes* and *Bacteroidota* between obese and lean dogs^15,17,44^. Park et al.^26^ reported that the relative abundance of *Firmicutes* was significantly lower in the obese group than that in the lean group, but no difference was observed in *Bacteroidota*. In a recent study, Macedo et al.^20^ showed a significantly higher proportion of *Bacteroidota* and a lower proportion of *Firmicutes* in obese dogs than that in lean dogs, which is similar to our findings. Therefore, a high relative abundance of *Firmicutes*, low relative abundance of *Bacteroidota*, and a high *Firmicutes*/*Bacteroidota* ratio might not be reliable biomarkers to identify obese dogs.

Butyrate is a major short-chain fatty acid in the gut environment because it is the main fuel for epithelial cells in the colon and exerts anti-inflammatory and anti-carcinogenic effects^45^; therefore, butyrate-producing bacteria are considered important microbiota^46^. In this study, *Faecalibacterium* and *Lachnospiraceae* NK4A136 group were enriched in the HBCS group. *Faecalibacterium* is the predominant genus belonging to Clostridial cluster IV and can produce butyrate and D-lactate by degrading a wide range of carbohydrate substrates^47^. Previous studies have reported conflicting results regarding the relative abundance of *Faecalibacterium* between obese and lean humans, such as a lower^48,49^ or higher^50–52^ abundance in obese humans. In the fecal microbiome of dogs, there are few reports on the relationship between members of *Faecalibacterium* and obesity. Recently, Macedo et al.^20^ reported that the relative abundance of *Faecalibacterium* was significantly higher in obese dogs than that in lean dogs, and it decreased significantly after the obese group underwent a weight loss program. Thus, further studies are needed to confirm the role of *Faecalibacterium* in dogs with obesity. *Lachnospiraceae* NK4A136 group is a potential butyrate producer^53^, and Companys et al.^52^ previously reported that the relative abundance of the *Lachnospiraceae* NK4A136 group was enriched in obese humans, which is supported by our results. Hence, the presence of butyrate-producing bacteria in the fecal microbiome of dogs may be related to obesity. The abundance of the family *Ruminococcaceae* was higher in the HBCS group. Many genera under *Ruminococcaceae* belong to cluster IV and possess enzymes that degrade recalcitrant carbohydrate sources and produce acetate as a major fermentation product that can be used as an energy source by the host^47^. In addition, four major genera related to acetate production as the major fermentation product, specifically *Fournierella*^54^, *Catenibacterium*^55^, *Megamonas*^56^, and unclassified genera within *Ruminococcaceae*^47^, were enriched in the HBCS group. *Faecalibacterium prausnitzii*, which is a major species belonging to *Faecalibacterium*, requires acetate for growth^57^. Therefore, the increase in *Faecalibacterium* may be linked to the high abundance of acetate-producing bacteria. Furthermore, several studies have suggested a relationship between *Sutterella* spp. and obesity in humans^58,59^. A previous study reported a higher relative abundance of *Sutterella* in obese dogs^20^, which is supported by our results. Thus, we speculate that the increase in *Sutterella* may be related to obesity in dogs. Serena et al.^60^ revealed that obesity in humans is associated with elevated levels of circulating succinate concomitant with specific changes in succinate-related microbiota, especially higher succinate producers (*Prevotellaceae* and *Veillonellaceae*) and lower succinate consumers (*Odoribacteraceae* and *Clostridiaceae*). In the present study, there were no significant differences in these families between the HBCS and NBCS groups. Instead, we found that the relative abundances of *Bacteroides*^61,62^ and *Mucispirillum*^63^, which are considered to be succinate producers^63^, and that of *Phascolarctobacterium*, which is a propionate producer through the succinate pathway, were higher in the HBCS group at the genus level. We thus suggest that obese dogs may have a higher relative abundance of succinate producers and succinate utilizer than lean dogs.

The alteration in fecal microbiota composition is linked to changes in microbial functional features. In this study, the difference in BCS did not affect the overall predicted functional features, as expected from the lack of differences in the overall bacterial diversity (Table 3). An excessive energy supply to the host by increasing carbohydrate metabolism is an important factor in obese animals^64^. In addition, several studies have reported an increase in the functional profile of carbohydrate metabolism in obese humans^65,66^. Therefore, we expected an increased relative abundance of functional profiles of carbohydrate metabolism in the HBCS group. However, carbohydrate metabolism among the top 10 KEGG categories showed a significant increase only in the NBCS group. Some KEGG modules involved in carbohydrate metabolism (galactose degradation [M00632], nucleotide sugar biosynthesis [M00549], and D-galactonate degradation [M00552]) were significantly enriched in the NBCS group. The unexpected increase in carbohydrate metabolism can be explained by two possible reasons: (1) the functional profile was predicted based on DNA; therefore, the RNA-based results (i.e.*.* RNA sequencing) in carbohydrate metabolism may be different from this result, and (2) the lack of a dog microbiome-specific PICRUSt2 database. In this study, some KEGG modules related to B vitamins biosynthesis (cobalamin biosynthesis [M00122] and pyridoxal-P biosynthesis [M00124]) and amino acid biosynthesis (lysine biosynthesis [M00030], serine biosynthesis [M00020], and tryptophan biosynthesis [M00023]) were enriched in the HBCS group. The microbiota produces many types of micronutrients that play an important role in host homeostasis^67^. B vitamins acts as an enzymatic cofactor or a precursor of cofactors^68^ and can be exchanged and shared between microbiota, thereby stabilizing the gut microbiota by cross-feeding^69^. A recent study that investigated the effects of co-culturing butyrate producers revealed that *Faecalibacterium prausnitzii*, a major species belonging to *Faecalibacterium*, requires several B vitamins and tryptophan for growth (auxotrophs)^70^. They also demonstrated that co-culturing *Faecalibacterium prausnitzii* with species belonging to *Lachnospiraceae*, which are prototrophic (capable of de novo synthesis) for all amino acids and several vitamins, stimulates the growth of *Faecalibacterium prausnitzii* through cross-feeding^70^. In this study, the HBCS group showed a higher relative abundance of butyrate producers (*Faecalibacterium* and *Lachnospiraceae* NK4A136 group) and obesity-related genera (*Lachnospiraceae* NK4A136 group and *Sutterella*). Turnbaugh et al.^25^ previously suggested that the microbiota of obese mice may be more efficient in energy harvesting than that of lean mice. Therefore, we speculate that obesity in dogs may be influenced by the enhancement of energy-harvesting efficiency through cross-feeding among fecal microbiota, especially among auxotrophs and prototrophs.

We also compared the overall interactions within the major fecal bacterial genera belonging to each BCS group. Unclassified genera within *Erysipelotrichaceae* and *Phascolarctobacterium* were the keystone genera within exclusive networks in the HBCS group. Previous studies have reported that several species belonging to the family *Erysipelotrichaceae* are associated with obesity in mouse models^71^ and humans^72^. Additionally, an association between *Erysipelotrichaceae* and host lipid metabolism has been suggested^73^. In the present study, UG_*Erysipelotrichaceae* had an exclusive positive interaction with five major genera, namely *Allobaculum*, *Fusobacterium*, *Lachnospiraceae* NK4A136 group, *Megamonas*, and *Phascolarctobacterium*. Among these genera, *Lachnospiraceae* NK4A136 group^52^ and *Megamonas*^74^, which were higher in abundance in the HBCS group, were also positively associated with obesity and weight gain, respectively. *Phascolarctobacterium* consumes succinate and produces propionate during carbohydrate fermentation^75^. Succinate is not only a metabolic end-product of some bacteria but also a primary cross-feeding metabolite in microbial propionate synthesis^76^. *Phascolarctobacterium* showed a strong positive correlation with 10 major genera, whereof three genera, namely *Fusobacterium*^77^, *Bacteroides*^61,62^, and *Mucispirillum*^63^ are considered to be succinate producers. Interestingly, *Muribaculaceae*, which showed no difference in the relative abundance between the HBCS and NBCS groups, exhibited exclusively negative correlations with four major genera, namely the *Lachnospiraceae* NK4A136 group, *Megamonas*, *Phascolarctobacterium*, and *Prevotellaceae* Ga6A1 group, which had significantly higher abundances in the HBCS group. Although little is known about the relationship between *Muribaculaceae* and obesity, a previous study revealed the resistance effect of *Muribaculaceae* in lean mice fed with a high-fat diet^78^. Therefore, we speculate that *Muribaculaceae* may play an anti-obesity role in dogs because of its negative correlation with obesity-related genera. In the exclusive network of the NBCS group, *Bifidobacterium* and *Negativibacillus* were the keystone genera. *Bifidobacterium* is well known as an important probiotic that exerts beneficial health effects, such as regulating microbial homeostasis, reducing intestinal lipopolysaccharide levels, and improving the mucosal barrier function^79–82^. In addition, several studies have reported that the relative abundance of *Bifidobacterium* is lower in mice fed a high-fat diet^83,84^. In this study, *Bifidobacterium* exhibited a strong negative correlation with *Peptoclostridium* and *Blautia*, which have previously been associated with obesity in dogs^20,85^, and a strong positive correlation with *Lactobacillus*, *Sellimonas*, and *Negativibacillus*. A previous study suggested that *Lactobacillus* may serve as a potential probiotic for dogs^27^ and *Sellimonas* plays an important role in gut recovery homeostasis^86^. Therefore, the co-occurrence interactions among potential probiotics and mutually exclusive interactions among obesity-related genera might have maintained the BW of the NBCS group.

The majority of previous studies investigating the fecal microbiome in obese dogs focused on the impact of dietary changes or physical training to reduce weight rather than on natural differences in the microbiome and microbial interactions. In this study, we investigated essential differences in the fecal microbiome and their interactions between normal and obese dogs. However, we found several results, especially regarding microbiota related to succinate metabolism and functional profiles of carbohydrate metabolism, that were inconsistent with the results of previous studies on obese mice and humans. Therefore, to better understand obesity in dogs, future studies should be conducted to further investigate the differences in microbial functional metabolism and metabolites between normal and obese dogs.

## Conclusions

Differences in BCS did not affect the overall distribution of the microbiota and predicted functional features. However, several genera related to short-chain fatty acid production were enriched in the HBCS group. In the predicted microbial functional profiles, some KEGG modules related to B vitamins and amino acid biosyntheses were higher in the HBCS group, indicating enhanced energy efficiency through cross-feeding between auxotrophs and prototrophs. In addition, specific co-occurrence and mutually exclusive interactions among the different genera were detected among the HBCS and NBCS groups. Further studies are needed to investigate how the specific networks can be interpreted in the context of fecal fermentation characteristics and obesity in dogs.

## Supplementary Information

Supplementary Information.

## Data Availability

The raw 16S rRNA sequences have been deposited in the NCBI SRA ubder BioProject PRJNA818097. All analyzed microbioal datasets in the present study are available from the corresponding author on reasonable request.

## Abbreviations

BW: Body weight
BCS: Body condition score
KEGG: Kyoto encyclopedia of genes and genomes
AAFCO: Association of American Feed Control Officials
QIIME2: Quantitative insights into microbial ecology 2
ASV: Amplicon sequence variant
ACE: Abundance-based coverage estimator
PCoA: Principal coordinate analysis
BIOM: Biological observation matrix
PICRUSt2: Phylogenetic investigation of communities by reconstruction of unobserved state
PCA: Principal component analysis
CoDiNA: Co-expression differential network analysis
PERMANOVA: Permutational multivariate analysis of variance
OUT: Operational taxonomic unit
