# Supplementary material for: Obese dogs exhibit different fecal microbiome and specific microbial networks compared with normal weight dogs
Source: Sci Rep. 2023 Jan 13;13:723. doi: 10.1038/s41598-023-27846-3 (PMC9839755; doi:10.1038/s41598-023-27846-3)
Supplement: Supplementary file 1 — Supplementary Information. [file 41598_2023_27846_MOESM1_ESM.docx]

# Supplementary data

Table S1. DADA2 denoising statistics of 16S rRNA gene amplicon sequencing variants between high and normal body condition score (BCS) groups.

| No. | Sex | BCS | BCS group^1^ | No. of input paired reads | Quality filtered reads | Denoised reads | Merged reads | Chimera- filtered reads | Taxa-filtered reads^2^ |
| --- | --- | --- | --- | --- | --- | --- | --- | --- | --- |
| 1 | Male | 8 | HBCS | 158,554 | 134,231 | 133,380 | 130,200 | 118,717 | 118,717 |
| 2 | Male | 9 | HBCS | 210,704 | 175,476 | 173,279 | 159,255 | 128,351 | 128,351 |
| 3 | Male | 7 | HBCS | 155,392 | 130,813 | 129,380 | 121,277 | 94,910 | 94,910 |
| 4 | Male | 7 | HBCS | 104,456 | 85,084 | 83,969 | 78,944 | 67,151 | 67,151 |
| 5 | Female | 9 | HBCS | 111,266 | 89,263 | 88,210 | 83,529 | 72,490 | 72,490 |
| 6 | Female | 8 | HBCS | 121,231 | 103,060 | 101,284 | 91,987 | 81,649 | 81,649 |
| 7 | Male | 4 | NBCS | 174,378 | 147,032 | 145,609 | 135,533 | 107,906 | 107,906 |
| 8 | Male | 6 | NBCS | 140,766 | 118,158 | 116,566 | 107,351 | 89,793 | 89,793 |
| 9 | Male | 5 | NBCS | 184,105 | 152,359 | 150,882 | 142,791 | 119,374 | 119,374 |
| 10 | Male | 5 | NBCS | 155,099 | 128,536 | 127,735 | 122,683 | 104,725 | 104,719 |
| 11 | Female | 5 | NBCS | 144,630 | 122,375 | 120,964 | 112,236 | 90,343 | 90,340 |
| 12 | Female | 5 | NBCS | 165,921 | 137,695 | 136,974 | 132,022 | 117,670 | 117,666 |

^1^ HBCS, high BCS group (BCS range: 7–9); NBCS, normal BCS group (BCS range: 4–6).

^2^ Amplicon sequencing variants identified as “Unassigned” were filtered out.


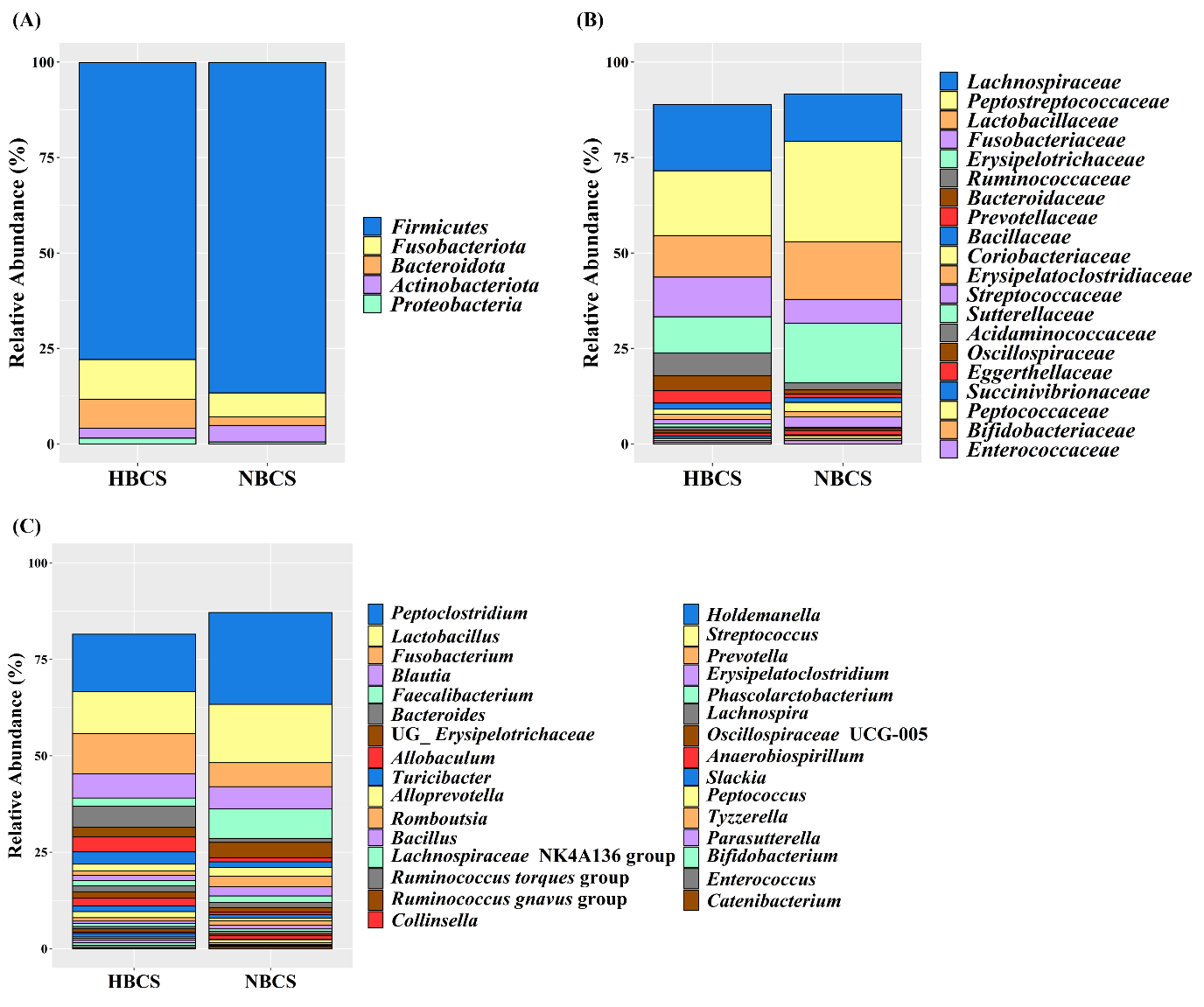


Figure S1. Distribution of the predominant fecal microbiota at (A) phylum, (B) family, and (C) genus levels. Taxa having a relative abundance ≥ 0.5% in at least one of the treatments are shown. HBCS, high body condition score (BCS) group (BCS range: 7–9, 4 males and 2 females, all 2-year-old); NBCS, normal BCS group (BCS range: 4–6, 4 males and 2 females, all 2-year-old); UG, unclassified genus within.


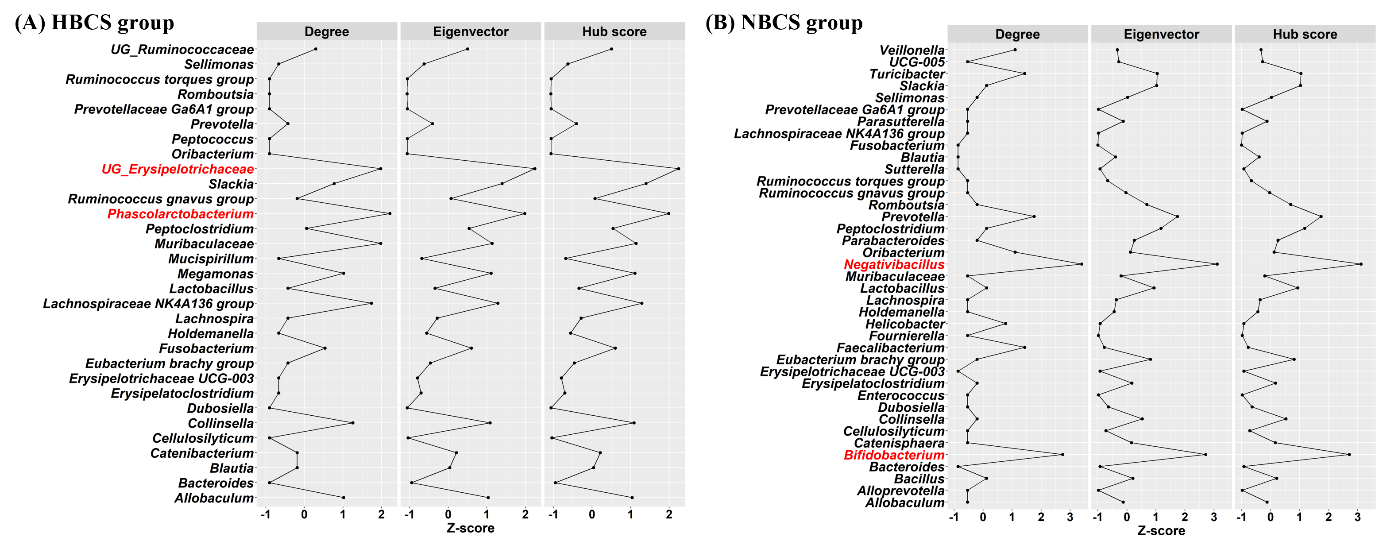


Figure S2. Centrality plots based on the degree centrality, eigenvector centrality, and hub score in (A) high body condition score group (HBCS; BCS range: 7–9, 4 males and 2 females, all 2-year-old) and (B) normal BCS group (NBCS; BCS range: 4–6, 4 males and 2 females, all 2-year-old). Only genera representing ≥ 0.05% abundance in at least one of the treatments were analyzed. Degree centrality, eigenvector centrality, and hub score were represented as Z-score, which was calculated as (raw value$-$mean)/standard deviation. Keystone genera (red color) were selected based on the top 5% taxa in terms of degree centrality, eigenvector centrality, and hub score.
